# Screening of bat faeces for arthropod-borne apicomplexan protozoa: *Babesia canis* and *Besnoitia besnoiti*-like sequences from Chiroptera

**DOI:** 10.1186/s13071-015-1052-6

**Published:** 2015-08-28

**Authors:** Sándor Hornok, Péter Estók, Dávid Kováts, Barbara Flaisz, Nóra Takács, Krisztina Szőke, Aleksandra Krawczyk, Jenő Kontschán, Miklós Gyuranecz, András Fedák, Róbert Farkas, Anne-Jifke Haarsma, Hein Sprong

**Affiliations:** Department of Parasitology and Zoology, Faculty of Veterinary Science, Szent István University, Budapest, Hungary; Department of Zoology, Eszterházy Károly College, Eger, Hungary; Department of Evolutionary Zoology and Human Biology, Debrecen University, Debrecen, Hungary; Centre for Infectious Disease Control, National Institute for Public Health and the Environment (RIVM), Bilthoven, The Netherlands; Plant Protection Institute, Centre for Agricultural Research, Hungarian Academy of Sciences, Budapest, Hungary; Institute for Veterinary Medical Research, Centre for Agricultural Research, Hungarian Academy of Sciences, Budapest, Hungary; Veterinary Authority, Miskolc, Hungary; Department of Animal Ecology and Ecophysiology, Radboud University Nijmegen, Nijmegen, The Netherlands

**Keywords:** Vector-borne, Chiroptera, Faecal DNA, Apicomplexa, *Dermacentor*, *Stomoxys*

## Abstract

**Background:**

Bats are among the most eco-epidemiologically important mammals, owing to their presence in human settlements and animal keeping facilities. Roosting of bats in buildings may bring pathogens of veterinary-medical importance into the environment of domestic animals and humans. In this context bats have long been studied as carriers of various pathogen groups. However, despite their close association with arthropods (both in their food and as their ectoparasites), only a few molecular surveys have been published on their role as carriers of vector-borne protozoa. The aim of the present study was to compensate for this scarcity of information.

**Findings:**

Altogether 221 (mostly individual) bat faecal samples were collected in Hungary and the Netherlands. The DNA was extracted, and analysed with PCR and sequencing for the presence of arthropod-borne apicomplexan protozoa. *Babesia canis canis* (with 99-100 % homology) was identified in five samples, all from Hungary. Because it was excluded with an Ixodidae-specific PCR that the relevant bats consumed ticks, these sequences derive either from insect carriers of *Ba. canis*, or from the infection of bats. In one bat faecal sample from the Netherlands a sequence having the highest (99 %) homology to *Besnoitia besnoiti* was amplified.

**Conclusions:**

These findings suggest that some aspects of the epidemiology of canine babesiosis are underestimated or unknown, i.e. the potential role of insect-borne mechanical transmission and/or the susceptibility of bats to *Ba. canis*. In addition, bats need to be added to future studies in the quest for the final host of *Be. besnoiti*.

## Findings

### Background

Microbats, known for their nocturnal activity and echolocation, belong to the second largest order (Chiroptera) of mammals and have a world-wide geographical distribution except arctic areas [1]. The great majority of their species are insectivorous, and therefore ecologically and economically important regulators of natural insect populations. Microbats also have a high epidemiological significance, due to their ability of “true flying” (frequently connected to migratory habit) and their presence in human settlements. In particular, roosting of bats in buildings (attics, cellars, stables) may bring pathogens of veterinary-medical importance into the environment of domestic animals and humans, thus increasing the chance of acquiring related infections. In this scenario bats have features that may further enhance their eco-epidemiological role, as exemplified by ubiquitous occurrence, long life-span, social behaviour (close contacts and allogrooming in colonies) and tendency for persistent infections [2].

Accordingly, bats are increasingly recognized as reservoirs or carriers (vectors) of various pathogen groups [3]. However, while numerous studies focused on emerging viruses (e.g. [2]) and bacteria (e.g. [4]) associated with bats, only a few recent, molecular surveys have been reported on their role as carriers of vector-borne protozoa [5, 6] – despite the close association of bats with arthropods (both in their food and as their ectoparasites [7]). Therefore, the present study was initiated to screen bat samples for arthropod-borne protozoa (Apicomplexa: Piroplasmida and related groups).

For this molecular survey bat faeces was chosen as the sample source, in part because of its non-invasive availability (that is a primary concern when handling small bodied, highly protected animal species). In addition, molecular investigation of bat faeces proved to be useful in taxonomical identification of macroscopic prey insects [8]. On the other hand, to the best of our knowledge, this method was hitherto not used to reveal the presence of arthropod-borne protozoa bats may have contact with. Demonstration of microbial/protozoan DNA from bat faeces is not only informative on prey insect (or bat intestinal) pathogens. It may also have relevance to the role bats may play as potential reservoirs of extraintestinal apicomplexans, because invasive stages or intracellular forms of these may cross the gut barrier. In this way the DNA of haemotropic protozoa may pass in detectable amounts with the faeces, as exemplified by *Plasmodium* spp. in primates [9].

## Methods

**Fig. 1:**
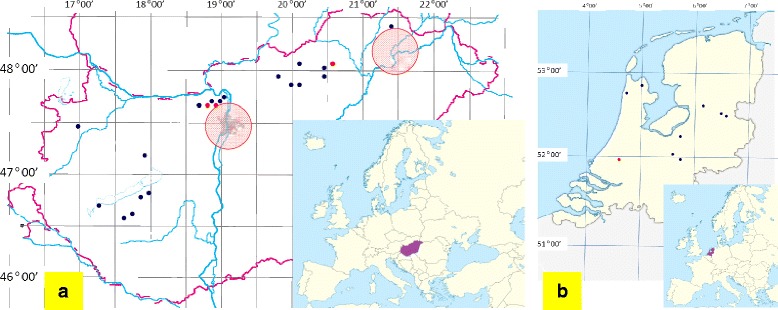
Map of Hungary (**a**) and Netherlands (**b**) showing the sampling sites. Only places at least 10 km apart are shown. The red dots on the map of Hungary (**a**) indicate places, where *Babesia canis* PCR positive bat droppings were collected. The shaded red circles mark the highly endemic regions of *Babesia canis* according to [16]. The red dot on the map of Netherlands (**b**) indicates the location, where the *Besnoitia besnoiti*-like sequence originated

Between May and September, 2014, 196 individual and 25 pooled bat faecal samples were collected (192 on 38 locations in Hungary, and 29 on 10 locations in the Netherlands: Fig. 1). The study involved the following 19 bat species (sample number): *Nyctalus noctula* (21), *N. leisleri* (9), *Myotis alcathoe* (23), *M. daubentonii* (49), *M. bechsteini* (21), *M. emarginatus* (6), *M. myotis* (8), *M. dasycneme* (4), *M. brandtii* (6), *M. nattereri* (13), *M. blythii* (5), *Rhinolophus ferrumequinum* (3), *R. hipposideros* (2), *Pipistrellus nathusii* (3), *P. pipistrellus* (14), *P. pygmaeus* (1), *Barbastella barbastellus* (6), *Miniopterus schreibersii* (1), *Plecotus auritus* (1). These bats were caught (as part of a monitoring program) at the entrance of caves between sunset and dawn, using standard Ecotone mist-nets (Gdynia, Poland) with 12 m length, 2.5 m height and 14 × 14 mm mesh. After identification the bats were individually held in sterile paper bags (i.e. one bat per one bag) until sufficient defecation. The standard sample size was three to five faecal pellets for each bat. The individual faecal pellets were transferred into numbered, screw cap plastic tubes and stored frozen at −20 °C until evaluation.

DNA was extracted with the QIAamp Fast DNA Stool Mini Kit (QIAGEN, Hilden, Germany) according to the manufacturer’s instructions and including extraction controls.

All samples were molecularly screened with a conventional PCR that amplifies an approx. 500 bp long part of the 18S rDNA gene of piroplasms [10]. This method also detects other apicomplexan genera, including vector-borne haemogregarines and certain cystogenic coccidia [11]. The primers BJ1 (forward: 5'-GTC TTG TAA TTG GAA TGA TGG-3') and BN2 (reverse: 5'-TAG TTT ATG GTT AGG ACT ACG-3') were used. The reaction volume was 25 μl, i.e. 5 μl of extracted DNA was added to 20 μl of reaction mixture containing 0.5 unit HotStarTaq Plus DNA polymerase (5U/ μl), 200 μM PCR nucleotid mix, 1 μM of each primer and 2.5 μl of 10× Coral Load PCR buffer (15 mM MgCl_2_ included). For amplification an initial denaturation step at 95 °C for 10 min was followed by 40 cycles of denaturation at 95 °C for 30 s, annealing at 54 °C for 30 s and extension at 72 °C for 40 s. Final extension was performed at 72 °C for 5 min.

Electrophoresis and visualization of the PCR product was done in a 1.5 % agarose gel, followed by sequencing (Biomi Inc., Gödöllő, Hungary). Representative sequences were deposited in the GenBank (accession numbers are shown in Table 1). Phylogenetic analyses were conducted according to the Tamura-Nei model [12] and Maximum Composite Likelihood method by using MEGA version 5.2 [13].

**Table 1:** Data of sample collections and results of molecular analyses according to country and bat species

Country	Date (2014)	Longitude	Latitude	Bat species (ring No.)	Results of sequencing (homology)	GenBank accession number
HUNGARY	July 19	20°33'06''	48°06'02''	*Nyctalus noctula*	*Babesia canis* (100 %)	KP835549
	August 29	18°52'30''	47°42'30''	*Myotis daubentonii* (A5783)	*Babesia canis* (100 %)	KP835549
	July 23	20°36'50''	48°06'39''	*Pipistrellus pygmaeus*	*Babesia canis* (99 %)	KP835550
	August 29	18°52'30''	47°42'30''	*Myotis daubentonii* (A5773)	*Babesia canis* (99 %)	KP835550
	August 30	18°50'35''	47°41'58''	*Myotis alcathoe*	*Babesia canis* (99 %)	KP835550
NETHERLANDS	July 28	4°39'05''	52°02'42''	Myotis dasycneme*	*Besnoitia besnoiti* (99 %)	KP835555

All except one (*) were individual samples. The reference sequences were FJ209024 for *Babesia canis* and KJ746531 for *Besnoitia besnoiti*. The bat ring number is also provided in the case of two samples collected from different individuals of the same bat species caught on the same date and in the same place

In addition, the presence of hard tick (Acari: Ixodidae) DNA in the bat faeces was evaluated by a conventional PCR that amplifies a 460 bp portion of the mitochondrial 16S rDNA gene of Ixodidae, with the forward primer 16S + 1 (5'-CTG CTC AAT GAT TTT TTA AAT TGC TGT GG-3') and reverse primer 16S-1 (5'-CCG GTC TGA ACT CAG ATC AAG T-3'). The original method [14] was slightly modified by using 1.0 unit of HotStartTaq Plus DNA polymerase in a reaction mixture as above, and a thermal profile of initial denaturation step at 95 °C for 5 min, followed by 40 cycles of denaturation at 94 °C for 40 s, annealing at 51 °C for 1 min, extension at 72 °C for 1 min, and final extension at 72 °C for 10 min.

Exact confidence interval (CI) for the prevalence rate was calculated at the 95 % level.

## Ethical approval

Authorization for bat capture was provided by the National Inspectorate for Environment, Nature and Water (No. 14/2138-7/2011). Bat banding licence numbers are TMF-14/32/2010 (DK) and 59/2003 (PE).

## Results and discussion

### *Babesia canis* DNA in bat faeces

**Fig. 2:**
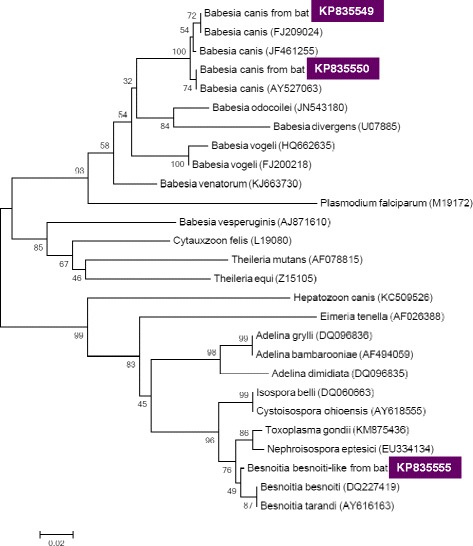
Phylogenetic comparison of 18S rDNA sequences of arthropod-borne apicomplexan protozoa identified in the present study (inverse colour), with related sequences from the GenBank. Branch lengths correlate to the number of substitutions inferred according to the scale shown

*Babesia canis canis* (referred to as *Ba. canis* onwards) DNA was shown to be present in five individual samples (prevalence 2.7 %, CI: 0.9-6.2 %), all from Hungary (Table 1). Two sequences were identified (accession numbers KP835549-50) with 2 nucleotide differences (inversion of GA to AG at positions 151–152 in the 18S rDNA gene). These bat-derived *Babesia* isolates showed 100 % identity with two *Ba. canis* isolates from dogs in Croatia (FJ209024 and FJ209025: [15]), and in phylogenetic comparison they clustered together with other *Ba. canis* isolates (Fig. 2). On the other hand, the relevant sequences exhibited only 88 % similarity to *Ba. vesperuginis* (AJ871610) known to infect bats (Fig. 2). All five bats with *Ba. canis* PCR positive faecal samples were caught within 50 km of the two regions in Hungary (Fig. 1), where the highest number of *Ba. canis* seropositive dogs were found in a previous countrywide survey [16].

Taken together, this may be the first molecular evidence that both main European genotypes of *Ba. canis* (group A, B: [17]) occur in Hungary.

There are three possible explanations for this unexpected finding. First, relevant bats may have eaten infected tick vectors of *Ba. canis*, i.e. *Dermacentor reticulatus*. To evaluate this possibility, the five *Babesia*-positive faecal DNA samples were molecularly analysed for the presence of tick DNA (mitochondrial 16S rDNA gene). All five samples were PCR negative. If relevant bats (with *Ba. canis* PCR positive faeces) have ingested infected tick vectors, the DNA of *D. reticulatus* should have been detected in their faeces, similarly to that of other prey arthropods [6]. This is supported by literature data: although bats also feed on arachnids, to the best of our knowledge ticks were never reported to be part of their diet (e.g. [8, 18]).

Alternatively, blood-sucking flies (e.g. *Stomoxys* spp.) are known to be incriminated as mechanical vectors in the transmission of *Babesia* spp. [19]. *Stomoxys calcitrans* (also called “dog fly”) was reported to frequently bite dogs [20], and to be a predominant species in the diet of some bat species [21]. Therefore, *Ba. canis* DNA in bat faeces may have originated from haematophagous flies which had sucked blood on parasitaemic dogs (in an opportunity offered by the two regions highly endemic for *Ba. canis*), and were consequently eaten by the relevant bats. Unfortunately, two factors precluded to test this hypothesis in the present study, i.e. (1) the whole faecal sample of relevant bats was used for DNA extraction (thus morphological analysis of fly remnants was not possible), and (2) to the best of our knowledge PCR-based molecular methods specific for *S. calcitrans* are not available.

However, the presence of *Ba. canis* DNA in the faeces may also indicate the infection of relevant bats (i.e. parasitaemia), in which case *Babesia* DNA could get from the circulation into the gut contents (similarly to the DNA of other erythrocyte-infecting protozoa, e.g. *Plasmodium* spp. in primates: [9]). In support of this possibility, among the preferred rodent hosts of *D. reticulatus* larvae/nymphs [22] many *Apodemus* spp. are arboreal, i.e. known for their climbing habit on trees [23]. *Dermacentor* larvae and nymphs were reported to be present in such arboreal nests [24], and in this way may be shared between rodents and bats [25]. All four bat species with *Ba. canis* PCR positive faeces (Table 1) are known for their preference of tree holes as summer roosting places [1, 26], where they could thus have become infested with *Dermacentor* larvae/nymphs (as reported for *Pipistrellus pipistrellus* sampled in July: [27]). Therefore, it cannot be completely excluded that those bats, which were PCR positive in their faeces, may have actually become infected with *Ba. canis* − a protozoan hitherto reported from two mammalian orders (besides Carnivora also from Perissodactyla: [28]), both taxonomically closely related to Chiroptera [29].

### *Besnoitia besnoiti*-like DNA in bat faeces

From one pooled faecal sample of a pond bat (*Myotis dasycneme*) colony roost in the Netherlands another sequence was identified, having the highest (99 %) homology with *Besnoitia besnoiti* (Table 1). The sequence (accession number KP835555) had six nucleotide difference from, but clustered together with *Be. besnoiti* and *Be. tarandi* (Fig. 2). It showed less (98 %) homology with (i.e. nine nucleotide difference) and clustered separately (Fig. 2) from a cystogenic coccidium, *Nephroisospora eptesici* recently identified from New World bats [30]. To the best of our knowledge, this is the first finding of a *Besnoitia*-like sequence from a non-ungulate mammal in Europe, and from any bat species in a world-wide context.

The source of the *Be. besnoiti*-like sequence in the present study, the pond bat (*Myotis dasycneme*) is known to be a long distant migratory species (up to 300 km seasonal migration: [31]), and the closest endemic focus of bovine besnoitiosis in northern France is situated within 300 km of the relevant sampling site [32]. In general, bats frequently use cattle stables for roosting [33], where they may have access to the mechanical vectors of *Be. besnoiti*, i.e. blood-sucking flies (*S. calcitrans*, *Tabanus* spp.) and mosquitoes [32]. In particular, *Tabanus* spp. and mosquitoes develop in wet soil near water and in water, respectively, corresponding to the main habitat of the pond bat. Blood-sucking flies (especially *S. calcitrans*) were also reported to constitute a significant portion of bat prey insects [21]. Therefore, the *Be. besnoiti*-like sequence in the present study might have originated from cattle via blood-sucking dipterans, or represents a novel *Besnoitia* genotype/species closely related to *Be. besnoiti*.

On the other hand, *Besnoitia* cystozoites (carried by flies) are able to penetrate mucosal surfaces [34]. Accordingly, the quest for the final host of *Be. besnoiti* should be extended to include chiropterans, particularly because experimental infection with another *Besnoitia* sp. was shown to establish in bats [35].

## Conclusions

These findings suggest that some aspects of the epidemiology of canine babesiosis are underestimated or unknown, i.e. the potential role of insect-borne mechanical transmission and/or the susceptibility of bats to *Ba. canis*. In addition, bats need to be added to future studies in the quest for the final host of *Be. besnoiti*.

In the present study no mixed infections were detected. This can be explained by the relatively low prevalence of those apicomplexans, the DNA of which could be amplified with the applied method [10] from bat faeces.

*Toxoplasma gondii* was reported to infect at least some of the bat species evaluated in the present study [36]. This apicomplexan is able to invade most nucleated cells (including cells crossing the gut barrier), and it was shown to be present in bat liver as well [37], therefore its DNA is likely to be shed in bat faeces. However, *T. gondii* was not detected in the present study. This can be explained by the inability of the applied method [10] to amplify toxoplasma DNA, because the forward primer BJ1 cannot anneal to the 18S rDNA gene of *T. gondii* with its 3' end, unlike in the case of piroplasms, *Besnoitia* and *Sarcocystis* spp. [11].

## References

[1] Dietz C, von Helversen O, Nill D (2009). Bats of Britain, Europe and northwest Africa.

[2] Calisher CH, Childs JE, Field HE, Holmes KV, Schountz T (2006). Bats: important reservoir hosts of emerging viruses. Clin Microbiol Rev.

[3] Klimpel S, Mehlhorn H (2014). Bats (Chiroptera) as vectors of diseases and parasites: facts and myths.

[4] Lin JW, Hsu YM, Chomel BB, Lin LK, Pei JC, Wu SH (2012). Identification of novel *Bartonella* spp. in bats and evidence of Asian gray shrew as a new potential reservoir of *Bartonella*. Vet Microbiol.

[5] Concannon R, Wynn-Owen K, Simpson VR, Birtles RJ (2005). Molecular characterization of haemoparasites infecting bats (Microchiroptera) in Cornwall, UK. Parasitology.

[6] Witsenburg F, Schneider F, Christe P (2014). Epidemiological traits of the malaria-like parasite *Polychromophilus murinus* in the Daubenton’s bat *Myotis daubentonii*. Parasit Vectors.

[7] Frank R, Kuhn T, Werblow A, Liston A, Kochmann J, Klimpel S (2015). Parasite diversity of European *Myotis* species with special emphasis on *Myotis myotis* (Microchiroptera, Vespertilionidae) from a typical nursery roost. Parasit Vectors.

[8] Hope PR, Bohmann K, Gilbert MT, Zepeda-Mendoza ML, Razgour O, Jones G (2014). Second generation sequencing and morphological faecal analysis reveal unexpected foraging behaviour by *Myotis nattereri* (Chiroptera, Vespertilionidae) in winter. Front Zool.

[9] Liu W, Li Y, Learn GH, Rudicell RS, Robertson JD, Keele BF (2010). Origin of the human malaria parasite *Plasmodium falciparum* in gorillas. Nature.

[10] Casati S, Sager H, Gern L, Piffaretti JC (2006). Presence of potentially pathogenic *Babesia* sp. for human in *Ixodes ricinus* in Switzerland. Ann Agric Environ Med.

[11] Hornok S, Mester A, Takács N, Baska F, Majoros G, Fok É (2015). *Sarcocystis*-infection of cattle in Hungary. Parasit Vectors.

[12] Tamura K, Nei M (1993). Estimation of the number of nucleotide substitutions in the control region of mitochondrial DNA in humans and chimpanzees. Mol Biol Evol.

[13] Tamura K, Peterson D, Peterson N, Stecher G, Nei M, Kumar S (2011). MEGA5: Molecular Evolutionary Genetics Analysis using Maximum Likelihood, Evolutionary Distance, and Maximum Parsimony Methods. Mol Biol Evol.

[14] Black WC, Piesman J (1994). Phylogeny of hard and soft-tick taxa (Acari: Ixodida) based on mitochondrial 16 s rDNA sequences. Proc Natl Acad Sci U S A.

[15] Beck R, Vojta L, Mrljak V, Marinculić A, Beck A, Zivicnjak T (2009). Diversity of *Babesia* and *Theileria* species in symptomatic and asymptomatic dogs in Croatia. Int J Parasitol.

[16] Hornok S, Edelhofer R, Farkas R (2006). Seroprevalence of canine babesiosis in Hungary suggesting breed predisposition. Parasitol Res.

[17] Adaszek L, Winiarczyk S (2008). Molecular characterization of *Babesia canis canis* isolates from naturally infected dogs in Poland. Vet Parasitol.

[18] Pereira MJR, Rebelo H, Rainho A, Palmeirim JM (2002). Prey selection by *Myotis myotis* (Vespertilionidae) in a Mediterranean region. Acta Chiropterol.

[19] Friedhoff, KT: Transmission of *Babesia*: Other means of transmission. In: Ristic M. (ed.) *Babesiosis of domestic animals and man*. CRC Press, Boca Raton, Florida; 1988. p. 45.

[20] Fankhauser B, Irwin JP, Stone ML, Chester S, Soll MD (2015). Repellent and insecticidal efficacy of a new combination of fipronil and permethrin against stable flies (*Stomoxys calcitrans*). Parasit Vectors.

[21] Kervyn T, Godin MC, Jocque R, Grootaert P, Libois R (2012). Web-building spiders and stable flies as prey of the notch-eared bat (*Myotis emarginatus*). Belg J Zool.

[22] Nosek J (1972). The ecology and public health importance of *Dermacentor marginatus* and *D. reticulatus* ticks in Central Europe. Folia Parasitol.

[23] Holišová V (1969). Vertical movements of some small mammals in a forest. Zool Listy.

[24] Durden LA, Hu R, Oliver JH, Cilek JE (2000). Rodent ectoparasites from two locations in northwestern Florida. J Vector Ecol.

[25] Apanaskevich DA, Bermúdez SE (2013). Description of a new *Dermacentor* (Acari: Ixodidae) species, a parasite of wild mammals in Central America. J Med Entomol.

[26] EUROBATS. Report on the implementation of ‘Eurobats’ in Hungary. *National report* 2010, 6:22. http://www.eurobats.org/sites/default/files/documents/pdf/National_Reports/nat_rep_Hun_2010.pdf [accessed March 3, 2015]

[27] Filippova NA, Neronov VM, Farhang-Azad A (1976). [Data on the ixodid fauna (Acarina, Ixodidae) of small mammals in Iran]. Entomologičeskoe Obozrenie.

[28] Hornok S, Edelhofer R, Földvári G, Joachim A, Farkas R (2007). Serological evidence for *Babesia canis* infection of horses and an endemic focus of *B. caballi* in Hungary. Acta Vet Hung.

[29] Nishihara H, Hasegawa M, Okada N (2006). Pegasoferae, an unexpected mammalian clade revealed by tracking ancient retroposon insertions. Proc Natl Acad Sci U S A.

[30] Wünschmann A, Wellehan JF, Armien A, Bemrick WJ, Barnes D, Averbeck GA (2010). Renal infection by a new coccidian genus in big brown bats (*Eptesicus fuscus*). J Parasitol.

[31] Limpens HJGA, Lina PHC, Hutson AM (2000). Action plan for the conservation of the pond bat in Europe (*Myotis dasycneme*).

[32] Alvarez-García G, Frey CF, Mora LM, Schares G (2013). A century of bovine besnoitiosis: an unknown disease re-emerging in Europe. Trends Parasitol.

[33] Dekker JJA, Regelink JR, Janssen EA, Brinkmann R, Limpens HJGA (2013). Habitat use of female Geoffroy’s bats (*Myotis emarginatus*) at it’s two northernmost maternity roosts and the implications for theur conservation. Lutra.

[34] Njenga MJ, Kang'ethe EK, Bwangamoi O, Munyua SJ, Mugera GM, Mutiga ER (1999). Experimental transmission of *Besnoitia caprae* in goats. J S Afr Vet Assoc.

[35] Schneider CR (1966). Experimental infection of short-tailed bats, *Carollia perspicillata*, with *Besnoitia panamensis* (Protozoa: Toxoplasmatidae). J Parasitol.

[36] Dodd NS, Lord JS, Jehle R, Parker S, Parker F, Brooks DR (2014). *Toxoplasma gondii*: prevalence in species and genotypes of British bats (*Pipistrellus pipistrellus* and *P. pygmaeus*). Exp Parasitol.

[37] Qin SY, Cong W, Liu Y, Li N, Wang ZD, Zhang FK (2014). Molecular detection and genotypic characterization of *Toxoplasma gondii* infection in bats in four provinces of China. Parasit Vectors.

